# ASA-score is associated with 90-day mortality after complicated mild traumatic brain injury – a retrospective cohort study, by Kiwanuka O et al.

**DOI:** 10.1007/s00701-024-06253-1

**Published:** 2024-09-17

**Authors:** Andreas Unterberg

**Affiliations:** University of Heidelberg, Im Neuenheimer Feld 400, Heidelberg, Germany

Recently, it became obvious that the epidemiology of traumatic brain injury (TBI) has changed in European countries during the last decades, mostly because of the aging of the population. Furthermore, mild TBI has attracted considerable interest, even among the neurosurgical community. Decades ago, when the distinction of mild, moderate and severe TBI was introduced on the basis of the Glasgow Coma Scale score, patients were significantly younger and only few patients with mild TBI had an abnormal CT, i.e. traumatic subarachnoid hemorrhage, subdural bleedings, contusions or else.

Nowadays, many patients with mild TBI are elderly, are hospitalized and experience a “complicated” mild TBI. It is not surprising that consequently, an increased mortality is seen.

The article by Kiwanuka et al. [1] focusses on Swedish TBI and trauma patients in a level III trauma center in Stockholm treated in 2019 who were identified using the Swedish Trauma registry.

The primary endpoint is 90-day mortality. The statistical analysis is looking for a better prognostication of death, particularly following mild TBI. For this reason, 244 patients with TBI were compared to 579 trauma patients without TBI.

The analysis as well some data need special comments:

1. The two groups are unevenly distributed. The many more non-TBI patients are younger and healthier. Not surprisingly, they have a (not significantly) lower mortality.
2. A (90-day) mortality of 8.2% in mild TBI patients may be regarded high. It may be not. In fact, mortality data in all grades of TBI have to be re-evaluated due to the changing epidemiology.
3. mTBI patients are elderly people and their pre-trauma ASA score is worse.

Mostly, they suffer from low-energy falls. Facts that were to be expected.

4. In both groups, deceased patients were older and had more comorbidities.

To be expected.

5. A closer look at the mortality of TBI patients, however, merits attention:
  Seven of 244 mild TBI patients (2,9%) die within their hospital stay, and 11 patients between 30- and 90-days post trauma (4,5%).
  To potentially prevent these deaths, one would like to know why they died in hospital and more so, why they died after having been discharged.
  Unfortunately, these questions are not answered by the article, since there are no data on reasons for death.
  Non-cerebral reasons for in-hospital deaths are most likely venous thrombosis, embolism, pneumonia etc. Reasons of death at home or in retirement/nursing homes are probably very different, e.g. a generally increased frailty, depression or maybe pure negligence of caregivers.
  Soon, the question arises whether relatives, caregivers or the society are truly willing to diminish the mortality of a disease that has formerly been regarded rather harmless.

Two further issues shall be touched, namely register studies and the ASA score.

Changing epidemiologies are strongly calling for register studies. The Swedish trauma register certainly is a good example. Traumatic brain injury (or neurotrauma in general), however is calling for more specific register that should include reasons of death and also more precise clinical outcomes.

The use of the ASA score as additional prognosticator is an interesting proposal. Although it may retrospectively be determined under certain circumstances, it may be debated whether it is widely used. The ASA score certainly is roughly estimating relevant comorbidities but is rarely available if prognostication or treatment decisions are of practical concern.

The mere association between ASA score and 90-day mortality in elderly patients with complicated mild TBI potentially enables better prognostication, but so far is of limited consequence. It would be, if respective patients would be followed more closely.

Do they, do we want this? This is a philosophical, this is a social question.
